# Metal–Organic Network-Based Composite Phase Change Materials with High Thermal and Photothermal Conversion Performance

**DOI:** 10.3390/ma18163814

**Published:** 2025-08-14

**Authors:** Dian Wei, Yi Wang, Shuoshuo Yu, Qingtang Zhang, Yi Wang

**Affiliations:** 1 College of Petrochemical Technology, Lanzhou University of Technology, Lanzhou 730050, China; wangyilut02@163.com (Y.W.); yushuo5896@163.com (S.Y.); zhqt137@163.com (Q.Z.); 2 State Key Laboratory of Advanced Processing and Recycling of Nonferrous Metals, Lanzhou University of Technology, Lanzhou 730050, China

**Keywords:** composite phase change materials, thermal energy storage, metal–organic networks, photothermal conversion

## Abstract

Solid–liquid phase change materials (PCMs), promising for thermal management, face limited application due to leakage and low thermal conductivity. In this work, a shape-stabilized composite PCM was fabricated using a one-pot in situ process by mixing polyethylene glycol (PEG) with the novel metal–organic network called CFK, which was synthesized from carboxylated multi-walled carbon nanotubes (CMWCNTs), FeCl_3_, and Kevlar nanofibers (KNFs). The morphology, composition, and thermophysical characteristics of the composite PCM were assessed. Key properties analyzed to validate its performance included leakage rate, thermal conductivity, latent heat, light absorption, photothermal conversion efficiency, and cycling stability. This composite PCM exhibits reduced leakage while maintaining remarkable thermal energy charge/discharge performance. The study establishes that the composite PCM containing 89.9 wt% PEG has a leakage rate of 0.76% since the PEG molecules are deeply embedded in the pores of CFK. The thermal conductivity of this composite PCM was enhanced by 170.5% relative to pure PEG, and the latent heat was measured as 147.9 J·g^−1^ for fusion and 143.7 J·g^−1^ for crystallization. Additionally, this composite PCM reveals excellent light absorption capacity, a photothermal conversion efficiency as high as 83.4%, and outstanding stability in photothermal cycling experiments. In short, this work offers a new strategy for both preparing high-performance composite PCMs and applying them in visible light conversion.

## 1. Introduction

Energy constitutes an essential material basis for the advancement of human society and functions as a pivotal driving force behind worldwide economic expansion. Seeking sustainable, safe, and clean energy solutions has become a global challenge. Thermal energy storage (TES) technology, by alleviating the contradiction between energy supply and demand, has attracted close attention from researchers [1,2]. Phase change materials (PCMs), as the core medium for storing latent heat, can store and discharge substantial thermal energy through isothermal phase transitions [3,4]. This capability of PCMs effectively improves energy utilization and serves a vital function in sustainable energy exploitation such as solar energy storage and application [5,6], thermal management of electronic devices [7], building insulation [8], and medical hyperthermia [9,10]. Among them, organic solid–liquid PCMs are most widely used in practical applications due to their advantages such as a wide phase change temperature, large phase change enthalpy, good chemical stability, and low price [11]. However, with the deepening of research, researchers have found that the enhanced fluidity of organic solid–liquid PCMs upon melting can easily lead to leakage [12]. When these materials remain in a liquid state under certain operating conditions, they not only fail to manage temperature effectively but may also exacerbate thermal runaway. More importantly, organic solid–liquid PCMs usually store latent heat through passive thermal absorption, which is highly susceptible to the external environment. Consequently, it is imperative to optimize traditional organic solid–liquid PCMs to possess both excellent stability and thermal energy storage performance.

Porous materials have been widely studied due to their regular pore structure and high specific surface area and have been applied in many fields such as guest molecule adsorption and separation, catalysis, energy storage, optoelectronics, sensing, and biomedicine [13,14]. Organic solid–liquid PCMs in the molten state can be efficiently adsorbed onto the pores or surface of porous, a process facilitated by the capillary forces within the porous matrix and intermolecular interactions. The resulting composite PCMs can maintain a stable shape during solid–liquid phase transitions [15]. Therefore, employing porous materials as matrices to prepare shape-stabilized PCMs has become a highly anticipated protocol [16,17]. This protocol usually uses traditional porous materials such as metal foam [18], carbon-based three-dimensional porous materials [19,20,21,22], and porous ceramics [23,24,25,26] as matrices to prepare composite PCMs. These matrices can efficaciously mitigate the leakage issue of the traditional PCMs and also improve their thermal conductivity to some extent. However, since most porous supports exhibit rigid frameworks and require solution impregnation or hot-melt vacuum injection for the preparation of composite PCMs, the resulting composites tend to be brittle. Additionally, they often exhibit poor interfacial contact with temperature-regulating surfaces, leading to significant interfacial thermal resistance.

Currently, novel porous materials called metal–organic networks have risen as ideal carriers for high-performance and multifunctional composite PCMs [27], owing to their tunable pore sizes, adjustable pore structures, and diverse chemical functionalities [28]. For example, Wang’s group developed a novel composite PCM based on metal–organic frameworks (MOFs), which exhibited photoluminescence-functionalized ability and significantly expanded the single thermal functionality of traditional PCMs [29]. While the micropores of MOFs effectively immobilize PCMs guest molecules, enhancing composite PCMs stability, the resulting nanoconfinement effect can inhibit PCMs crystallization, compromising latent heat release [30]. These factors cause the phase transition enthalpy of traditional MOF-based composite PCMs to be well below pure PCMs. Recently, Pan’s group prepared multilayer structure porous support material PVP@Co_3_O_4_/EG by crosslink of polyvinyl pyrrolidone. The addition of octadecanol to prepare the composite phase change material MOF-EG/OC resulted in a phase transition enthalpy of 187.04 J·g^−1^ [31]. Kim’s group synthesized the high topology (3,6)-connected Zn-MOFs as support materials that were used to enhance the phase transition enthalpy of composite PCMs [32]. Wang’s work demonstrates that the hierarchical rGO@MOF-5-C achieves a latent heat of 168.7 J·g^−1^, representing an 18.5% increase compared to conventional non-hierarchical matrix materials [33]. These works have enhanced the thermal performance of PCMs by introducing MOF-based matrix to some extent. This indicates that the thermal storage performance of composite PCMs can be optimized by adjusting the pore size and porosity of the matrix materials [34,35,36]. The phase transition enthalpy of the composite PCMs increases with the amount of adsorbed pure PCMs. On the other hand, numerous studies demonstrate that the active sites of support materials can be increased by introducing heteroatoms, thereby enhancing the supporting ratio of support materials and further improving the thermal storage performance of composite PCMs [37,38,39,40]. Although the support materials of PCMs mentioned in the above studies have achieved significant progress in enhancing thermal storage performance and shape stability, they are often plagued by obstacles such as non-adjustable pore sizes, complex fabrication, and poor elasticity. Furthermore, research on existing support materials remains insufficient for multifunctional PCMs. Thus, developing porous support materials with tunable pore sizes and photothermal conversion capabilities through facile preparation methods is crucial for fabricating high-performance composite PCMs. These materials not only ensure excellent thermal storage performance and guest molecule compatibility but also provide traditional PCMs with multi-pathway thermal storage capabilities.

Based on the above studies and our long-term interest in composite PCMs [41,42,43], we propose a strategy to prepare support materials featuring high porosity and multiple active sites by utilizing substrates with multi-microporous structures and abundant heteroatoms. Specifically, a novel three-dimensional metal–organic network (CMWCNT/Fe^3+^/KNFs, abbreviated as CFK) was constructed through the interaction between Fe^3+^ and carboxyl groups in CMWCNT (carboxylated multi-walled carbon nanotubes) featuring microporous structures and multiple active sites, using nitrogen-rich KNFs (Kevlar nanofibers) as support enhancer. This shape-stabilized composite PCM was prepared in one pot by loading PEG (polyethylene glycol) into CFK directly without post-treatment. The stability, morphology, chemical structure, and thermal performance of the composite PCM was characterized and analyzed in detail. This composite PCM exhibits satisfactory shape stability, high phase transition enthalpy, and high thermal conductivity. Furthermore, this work explored the photothermal conversion performance of this material under simulated sunlight. This composite PCM material, with excellent shape stability, heat storage performance, and photothermal conversion performance, demonstrates promising application potential in the field of solar energy storage.

## 2. Materials and Methods

### 2.1. Materials

Analytical grade PEG (molecular mass 4000), dimethyl sulfoxide, and potassium hydroxide were supplied by Aladdin. Kevlar nanofibers (KNFs) were sourced from Changzhou Hualike New Material Co., Ltd. (Changzhou, China). FeCl_3_·6H_2_O (analytical grade) and triethylamine (TEA, analytical grade) were acquired from Yantai Shuangshuang Chemical Co., Ltd. (Yantai, China). Carboxylated multi-walled carbon nanotubes (CMWCNT, purity: 95%, carboxyl content: 2.56 wt%) were supplied by Nanjing XFNANO Materials Tech Co., Ltd. (Nanjing, China). Anhydrous ethanol was procured from Sinopharm Chemical Reagent Co., Ltd. (Shanghai, China). Deionized water sourced from the laboratory’s purification system was employed as the solvent.

### 2.2. Synthesis of PEG/CFK Composite PCMs

The PEG/CFK composite phase change material was prepared with a simple one-pot method using FeCl_3_·6H_2_O and CMWCNT as raw materials to coordinate and form a cross-linked porous matrix, with PEG serving as the PCM and KNFs introduced as support reinforcing agents. In this nomenclature, the letters C, F, and K represent CMWCNT, FeCl_3_·6H_2_O and KNFs, respectively.

**Figure 1:**
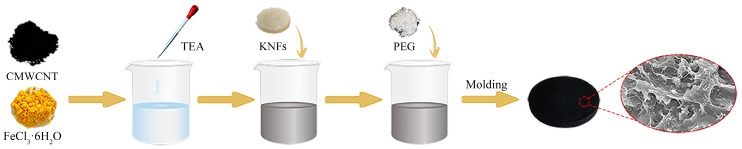
The schematic illustration of PEG/CFK preparation process.

Specifically, following the dispersion of CMWCNT (0.2 g) in anhydrous ethanol (15 mL), the mixture of FeCl_3_·6H_2_O (0.15 g), TEA (0.3 g), and wet KNF (0.1 g) gel was added. After stirring evenly, a predetermined amount of PEG-4000 was added, and the system was magnetically stirred at 70 °C for 4 h. Finally, the resulting mixture was poured into a mold and dried under forced air at 80 °C to remove the anhydrous ethanol and TEA. A schematic illustration of the construction route is presented in Figure 1. PEG mass fraction affects the composite’s shape stability and heat storage performance. A low PEG mass fraction results in low thermal storage density, while an excessively high PEG content can easily lead to leakage. Based on this, we designed a solution that keeps the amount of support material constant while adjusting the amount of PEG in order to find the optimum PEG ratio. The composite PCMs were labeled as PEG/CFK-x (x = 1, 2, 3) based on the predetermined mass fraction of PEG, with their corresponding compositions summarized in Table 1.

### 2.3. Characterization

Visual inspection method and gravimetric analysis was applied to assess macroscopic morphological changes of composite PCMs and pure PEG at different temperatures above the phase transition temperature of PEG. The leakage rate of PEG/CFK was evaluated by measuring the mass change before and after heating in an 80 °C oven for 1 h.

The microstructure of the PEG/CFK was examined using scanning electron microscopy (SEM). Characterizations were performed using a Carl Zeiss Sigma 300 instrument (Baden-Württemberg, Oberkochen, Germany) operating at an accelerating voltage of 3 kV. Before imaging, the materials were preprocessed by lyophilizing, grinding, and mounting on conductive adhesive.

Fourier transform infrared (FTIR) (Nicolet 6700, Thermo Nicolet Corporation, Waltham, MA, USA) served to examine the chemical composition of composite PCMs. Spectral data covering 400 cm^−1^ to 4000 cm^−1^ were acquired at 4 cm^−1^ resolution under ambient conditions.

X-ray diffraction (XRD) was applied to the analysis of the composites by utilizing Bruker D8 Advance diffractometer (Bavaria, Karlsruhe, Germany). The device was operated by utilizing a Cu K*α* source (λ = 0.154 nm) at 40 kV and 40 mA, with output acquisition spanning 5° to 60° using a scan rate of 2°·min^−1^.

The heat endurance of composites was tested by utilizing a thermogravimetric analysis (TGA) instrument (TGA2, Mettler-Toledo International Inc., Greifensee, Switzerland). Measurements were carried out under a flow of N_2_ (50 mL·min^−1^) with a constant heating rate of 10 °C·min^−1^, scanning from 35 °C to 800 °C.

The thermal conductivity of the compacted composite materials was evaluated using a thermal conductivity (TC) meter (TC3200, XIATECH, Xi’an, China) at ambient conditions. To obtain reliable results, each sample underwent quintuplicate parallel analysis.

The phase transition enthalpy ΔH and temperature of samples were obtained using a differential scanning calorimetry (DSC, DSC3, Mettler-Toledo International Inc., Greifensee, Switzerland) under a flow of N_2_ (50 mL·min^−1^) with a constant heating rate of 3 °C·min^−1^. Prior to testing, the instrument’s temperature was elevated from ambient temperature to 80 °C and then restored to ambient temperature to erase its thermal history influences.

The photothermal conversion ability of the PEG/CFK was assessed at ambient temperature using a xenon lamp to simulate solar radiation. During the testing process, the light intensity was fixed at 100 mW·cm^−2^, and the platform height was adjusted to position the sample 15 cm from the lamp. The real-time temperature on the material’s upper surface was monitored using a thermal imager (H30, Tianbo Cloud-Tech, Hangzhou, China). Based on the measured temperatures, corresponding time–temperature curves were plotted. Within the temperature rising interval, the time corresponding to the formation of the phase change plateau is denoted as T_0_, while the time at the end of the phase change plateau is recorded as T_t_. The photothermal conversion efficiency, η, was determined using formula (1) based on a previous reference [44]:

(1) $$\eta =\frac{m\Delta H}{PS(T_{t}-T_{0})}$$

where m is sample mass (g), ΔH denotes the latent heat of fusion (J·g^−1^), P represents incident irradiance (100 mW·cm^−2^) derived from the xenon lamp, S corresponds to the sample surface area (cm^2^), and T_0_ and T_t_ denote the initial and final time of the phase transition process, respectively.

## 3. Results and Discussion

### 3.1. Determination of the Composition and Shape of PEG/CFK

**Figure 2:**
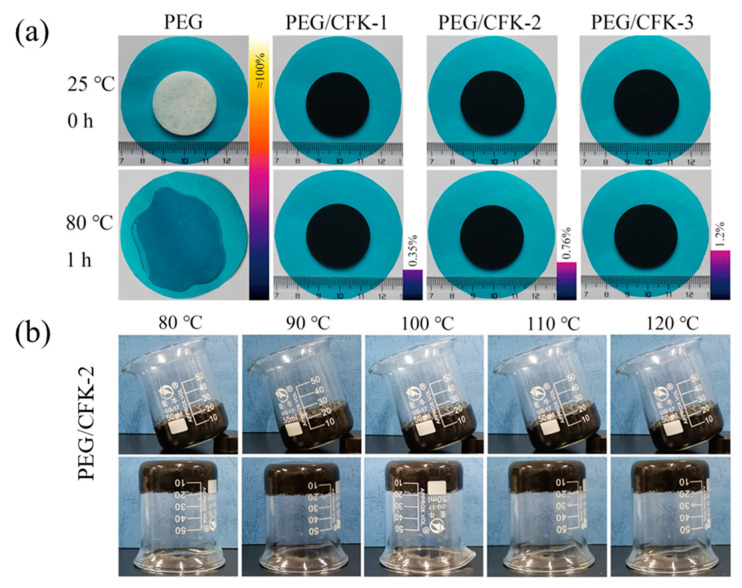
(**a**) Leakage rate test of pure PEG and composite phase change materials PEG/CFK in 80 °C; (**b**) Shape stability of PEG/CFK-2 at different temperatures.

The PEG content in CFK is critical to the thermal energy storage capacity, service life, and cost-effectiveness of the composite PCMs. To explore the optimal composition of PEG/CFK, samples with PEG mass fractions of 87%, 89%, and 91.7% were synthesized, designated as PEG/CFK-1, PEG/CFK-2, and PEG/CFK-3, respectively. The pure PEG and the above samples were subjected to leakage testing by heating at 80 °C for 1 h. Composite PCMs with a leakage rate below our specified limit of 1% and maintained shape stability were acceptable. The experimental results revealed that the pure PEG completely melted, whereas all PEG/CFK samples retained their original morphology, demonstrating the CFK framework’s effective restriction of PEG molecules. Furthermore, the leakage rates of PEG/CFK-1, PEG/CFK-2, and PEG/CFK-3 progressively increased with higher PEG content. Considering the leakage rate, PEG loading, and shape stability collectively, PEG/CFK-2 with a leakage rate of 0.76% was selected as the optimal material (Figure 2a).

To further investigate the shape stability, the partially dried PEG/CFK-2 samples were sequentially exposed to temperatures from 80 °C to 120 °C (in 10 °C increments), with findings displayed in Figure 2b. The outcomes demonstrated that PEG/CFK-2 remained stationary and without detachment from the beaker under both tilting and inverted conditions. This is attributed to the effective confinement of the metal organic framework on PEG molecules, enabling PEG/CFK-2 to maintain shape stability even at relatively elevated temperatures.

### 3.2. Morphology and Structure of CFK and PEG/CFK-2

**Figure 3:**
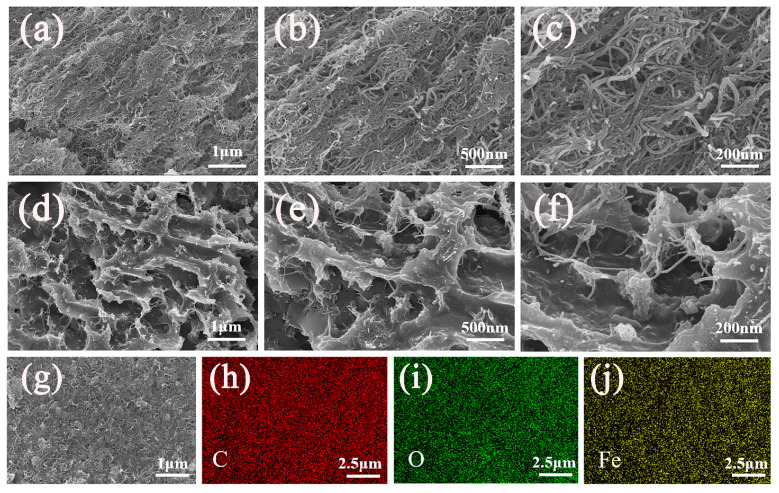
(**a**–**c**) SEM images of CFK; (**d**–**f**) SEM images of PEG/CFK-2; (**g**) SEM and (**h**–**j**) EDS images of CFK.

The surface morphology and microstructure of microcapsules of PEG/CFK-2 and carrier materials were characterized using SEM. In Figure 3a,b, the synthesized CFK carrier material exhibits a characteristic porous layered structure, which is likely formed through interactions between carboxyl groups and Fe^3+^ ions. Moreover, hydrogen bonding interactions between the Kevlar nanofibers and CMWCNT may further strengthen this porous network structure. Figure 3c clearly demonstrates that the carrier material CFK retained the original tubular structure of CMWCNT, exhibiting a curled and entangled morphology. As illustrated in Figure 3d–f, the composite material formed by incorporating PEG into CFK exhibits an uneven surface and robust pore channels. This morphology is likely attributed to hydrogen bonding interactions between PEG molecules and CFK. The above results also indicate that PEG is effectively encapsulated in the support material. To further confirm the structure of CFK, the energy dispersive spectrometer (EDS) characterization were conducted (Figure 3g–j). The results indicate that Fe^3+^ ions were uniformly distributed in CFK. The above results correlate with the formation of a three-dimensional metal–organic network in CFK involving interactions between CMWCNT and Fe^3+^ ions.

**Figure 4:**
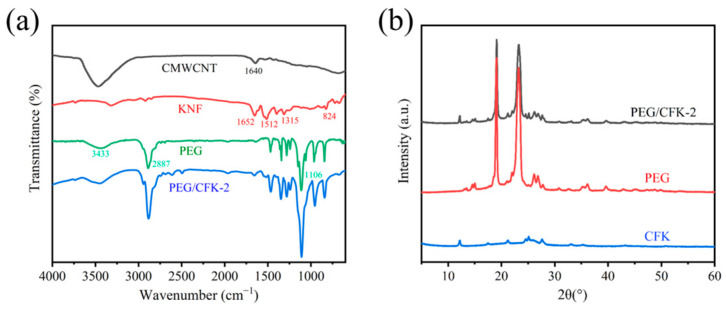
(**a**) FTIR spectrums of CMWCNT, KNF, PEG, and PEG/CFK-2; (**b**) XRD patterns of PEG/CFK-2, PEG, and KNF.

Subsequently, the chemical structure of PEG/CFK-2 was explored using FTIR (Figure 4a). The FTIR spectrum of pure PEG exhibited a broad O-H stretching vibration at 3433 cm^−1^ and a strong C-O stretching vibration at 2887 cm^−1^ [45]. For pure CMWCNT, its spectrum exhibited a characteristic peak at 1640 cm^−1^ owing to stretching vibrations of C=O bonds [46]. The characteristic peak for KNFs, such as stretching vibrations of C=O bonds at 1652 cm^−1^, stretching vibrations of C(sp^2^)-C(sp^2^) bonds of the benzene ring at 1512 cm^−1^, stretching vibrations of C-N bonds at 1315 cm^−1^ in the amide fragment, and stretching vibrations of C-N bonds at 824 cm^−1^ in the aniline fragment, were presented [47]. Furthermore, there are no new peaks appeared in the spectrum of PEG/CFK-2 compared to the pure components mentioned above. These results indicated that PEG/CFK-2 was formed by the physical binding of PEG and other components without chemical change. As Figure 4b shows, the XRD patterns of PEG/CFK-2 showed diffraction peaks at 19.1° and 23.2° that matched the characteristic diffraction peak angles of pure PEG [48]. This further indicated that PEG/CFK and PEG had similar crystal structures and unit cell types. However, the diffraction intensity of PEG/CFK-2 decreased compared with pure PEG. The decline may be attributable to the network structure formed by carboxylated MWCNTs, Fe^3+^, and KNFs, which limited the crystallization of PEG.

### 3.3. Thermodynamic Properties of PEG/CFK-2

**Figure 5:**
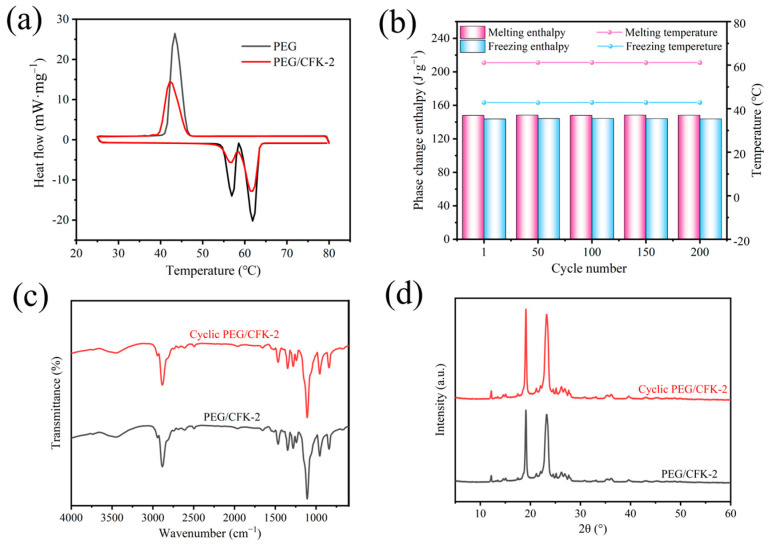
(**a**) DSC curves of PEG and PEG/CFK-2; (**b**) Graphic of enthalpy and temperature for phase transition in PEG/CFK-2 at every 50 cycles; (**c**) FT-IR spectrum and (**d**) XRD of PEG/CFK-2 after 200 thermal cycles.

The DSC results of PEG/CFK-2 and PEG are presented in Figure 5a. Table 2 illustrates the thermophysical parameters of melting (m) and crystallization (c) processes that stemmed from DSC, including onset temperature (T_om_ and T_oc_), peak temperature (T_pm_ and T_pc_), end temperature (T_em_ and T_ec_), and phase transition enthalpy (ΔH_m_ and ΔH_c_). Here, the melting point (61.1 °C) and freezing point (42.8 °C) of PEG/CFK-2 were very close to those of PEG (61.2 °C and 44.1 °C, respectively), and their DSC curve trends were highly consistent. It was indicated that the thermal energy performance of PEG/CFK-2 was primarily derived from the PEG component. Careful observation reveals that the onset melting temperature (T_om_) of the composite material exhibits a slight decrease compared to pure PEG, which may be caused by the difference in thermal conductivity between the two materials. Moreover, the melting and freezing enthalpies of PEG/CFK-2 were 147.9 J·g^−1^ and 143.7 J·g^−1^, accounting for 87.7% and 88.9% of those of pure PEG, respectively. It was clear that PEG/CFK-2 presented excellent thermal storage performance. In addition, the latent heat of the composite PCM shows a certain decrease compared to that of the pure PCM. This suggests a corresponding reduction in the crystallinity of PEG/CFK-2, a trend also consistent with the observations from XRD. The thermal cycling stability of PCMs is a core factor in their practical applications. The enthalpy and peak temperature of PEG/CFK-2 after different cycles are shown in Figure 5b and Table 3. It was clear that, after 50, 100, 150 and 200 DSC cycles, PEG/CFK-2 displayed no significant changes in its melting and freezing enthalpy, and the change in phase transition temperature was also insignificant. Moreover, there were no new absorption peaks observed in the FTIR spectrum and XRD patterns of PEG/CFK-2 after thermal cycling test, suggesting that the chemical structure of PEG/CFK-2 was stable under thermal cycling (Figure 5c,d). In short, PEG/CFK-2 shows good thermal cycling stability, which meets the requirements for repeated practical use.

**Figure 6:**
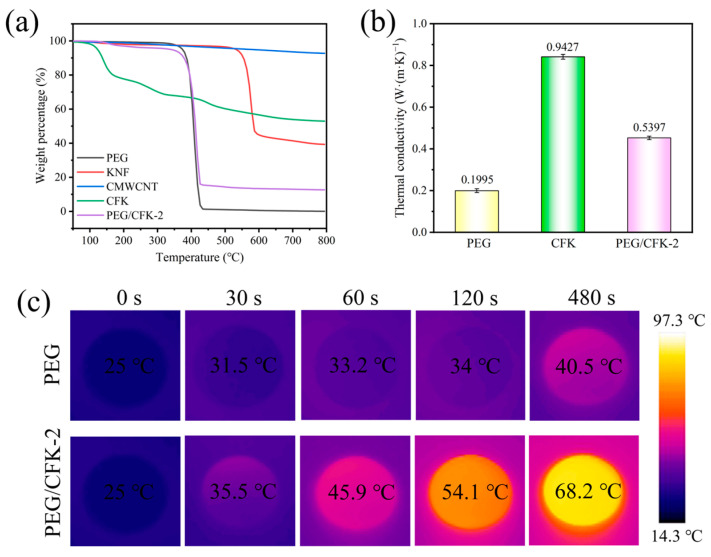
(**a**) TGA curves of PEG/CFK-2 and raw materials; (**b**) Thermal conductivity of PEG, CFK, and PEG/CFK-2; (**c**) Thermal infrared imaging of PEG and PEG/CFK-2.

To evaluate the chemical stability of PEG/CFK-2, its decomposition temperature was measured using TGA. As shown in Figure 6a, pure PEG was decomposed in a single-step between 351 °C and 433 °C, with a weight loss of 99.9%. CMWCNT exhibited excellent thermal stability, losing only 7.1% of its weight at 800 °C. The degradation temperature range of KNF was 520 °C to 600 °C, with a mass loss of 56% over the degradation process. In the weight loss curve of CFK, the weight loss in 100 °C to 200 °C and in 200 °C to 400 °C corresponded to the loss of FeCl_3_·6H_2_O, and the final stage corresponded to the loss of KNF. The total weight loss for the entire process was 46.7%. The degradation process of PEG/CFK-2 presented with three stages, with a total weight loss of 87.3%. Compared with the weight loss curve of CFK and PEG, it can be concluded that the first and third stages corresponded to the decomposition of CFK, although the weight loss in these stages was negligible due to the low CFK content. Obviously, the second stage with the most significant weight loss was caused by the degradation of PEG. The above results indicated that PEG/CFK-2 possessed a high degradation temperature and good chemical stability.

The low thermal conductivity (≤0.2 W·m^−1^·K^−1^) of pure PEG poses a bottleneck that severely restricts its thermal energy charge/discharge rates, which has become a key application challenge for this class of materials. Incorporating high thermal conductivity fillers into PCMs to prepare composites is an effective method to enhance their thermal performance. Here, the enhancement effect of CFK on the thermal conductivity of composites was tested (Figure 6b). The results indicated that the incorporation of CFK increased the thermal conductivity of the composites to 0.5397 W·m^−1^·K^−1^, representing a 170.5% enhancement compared to pure PEG. Thus, CFK improved the thermal conductive performance of the composites. It is noteworthy that the high thermal conductivity of composite materials may lead to a slight reduction in T_om_ compared to low thermal conductivity PEG, which aligns with the aforementioned DSC results. The enhanced high thermal conductivity offers more significant advantages for phase-change materials in thermal management applications, including efficient heat transfer, temperature uniformity, energy conservation, and consumption reduction. Moreover, considering the photothermal conversion capability of CFK, we further investigated its impact on the heating rate of composite materials under light irradiation (Figure 6c). Thermal infrared imaging clearly demonstrated that PEG/CFK-2 exhibited a higher surface temperature than PEG under identical starting temperatures and light. Apparently, PEG/CFK-2 demonstrated a higher heating rate, which was consistent with the tested thermal conductivity results.

### 3.4. Photothermal Conversion Performance of PEG/CFK-2

**Figure 7:**
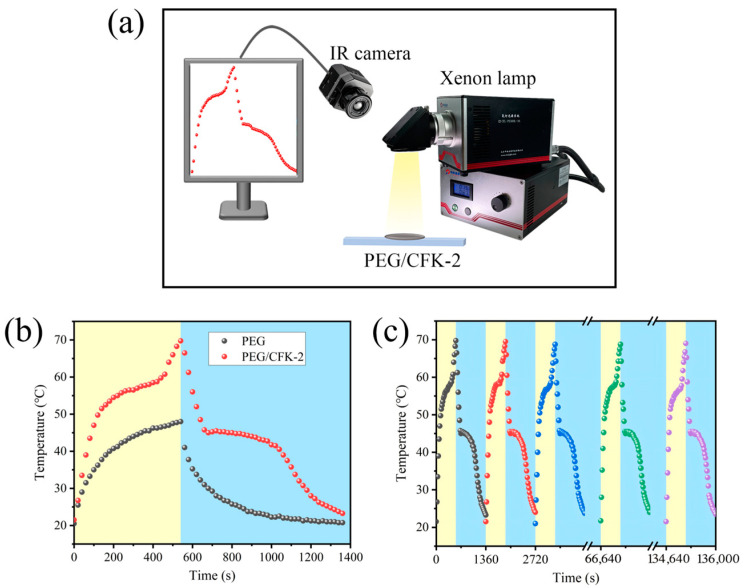
(**a**) Solar simulator; (**b**) Temperature–time curves of PEG and PEG/CFK-2 under a light; (**c**) Temperature–time curve for 100 cycles of PEG/CFK-2.

The photothermal conversion performance of PEG/CFK-2 was investigated using a solar simulator under a light intensity of 100 mW·cm^−2^ (Figure 7a), and the temperature–time curve is presented in Figure 7b. After the xenon lamp was turned on, the surface temperature of the PEG/CFK-2 composite rapidly rose to near the melting point of PEG, followed by a slowed heating rate and the appearance of a phase transition plateau. This was because the PEG in PEG/CFK-2 stores the heat converted from luminous energy in the form of latent heat during the solid–liquid phase change. After the phase change process was completed, the surface temperature of PEG/CFK-2 continued to rise to 70.5 °C with prolonged illumination time, with the whole heating stage lasting for 540 s. After turning off the xenon lamp, the surface temperature of PEG/CFK-2 first decreased rapidly, followed by a distinct latent heat release plateau. During this stage, the cooling rate slowed down due to the release of latent heat, with the plateau persisting for approximately 360 s. Finally, the PEG/CFK-2 gradually returned to its initial temperature. However, the temperature of pure PEG before and after light exposure remained lower than that of PEG/CFK-2 under identical conditions, with a peak temperature of only 48 °C. In addition, the photothermal conversion efficiency of PEG/CFK-2 was calculated to be 83.4% using formula (1). The above results suggested that PEG/CFK-2 effectively converted luminous energy into thermal energy and stored it in the form of latent heat. Then, PEG/CFK-2 was subjected to cyclic experiments under identical conditions to evaluate the photothermal cycling stability. As shown in Figure 7c, the temperature–time curves of PEG/CFK-2 before and after 100 cycles were nearly identical, indicating excellent photothermal cycling stability of PEG/CFK-2.

## 4. Conclusions

In summary, a three-dimensional metal–organic network CFK was prepared using simple raw materials with a one-pot reaction, and PEG was encapsulated within it to fabricate a novel composite phase change material PEG/CFK. PEG and the CFK support were bound through intermolecular interactions in this material, which enabled it to exhibit good compatibility and shape stability at 120 °C with a leakage rate as low as 0.76%. The PEG/CFK-2 with 89.9 wt% PEG presented excellent thermal storage capacity and cycling stability, demonstrating enthalpies of melting and solidification of 147.9 J·g^−1^ and 143.7 J·g^−1^, respectively. Even after 200 uninterrupted thermal cycles, both its enthalpy values and phase change temperatures remained unchanged. Compared to pure PEG, the thermal conductivity of PEG/CFK-2 showed a noticeable improvement. On the other hand, PEG/CFK-2 exhibited excellent photothermal conversion performance and cycling stability with an efficiency of 83.4% and retained its performance after 100 photothermal cycles. The design protocol of this work provided an important reference value for the study of composite PCMs with high loading, stable shape, and photothermal conversion performance.

## Figures and Tables

**Table 1:** Raw material ratio for PEG/CFK preparation.

Sample	FeCl_3_·6H_2_O	CMWCNT	KNFs	PEG	ω (PEG)
	(g)	(g)	(g)	(g)	(%)
PEG/CFK-1	0.15	0.2	0.1	3.0	87.0%
PEG/CFK-2	0.15	0.2	0.1	4.0	89.9%
PEG/CFK-3	0.15	0.2	0.1	5.0	91.7%

**Table 2:** Thermophysical properties data of PEG and PEG/CFK-2.

	Melting Process				Crystallization Process			
Samples	T_om_	T_pm_	T_em_	ΔH_m_	T_oc_	T_pc_	T_ec_	ΔH_c_
	(°C)	(°C)	(°C)	(J·g^−1^)	(°C)	(°C)	(°C)	(J·g^−1^)
PEG	59.3	61.2	63.2	168.6	46.3	44.1	41.6	161.7
PEG/CFK-2	58.7	61.1	63.4	147.9	46.5	42.8	40.1	143.7

**Table 3:** Thermophysical parameters of PEG/CFK-2 at every 50 cycles.

	Melting Process				Crystallization Process			
Cycle Number	T_om_	T_pm_	T_em_	ΔH_m_	T_oc_	T_pc_	T_ec_	ΔH_c_
	(°C)	(°C)	(°C)	(J·g^−1^)	(°C)	(°C)	(°C)	(J·g^−1^)
1	58.7	61.1	63.4	147.9	46.5	42.8	40.1	143.7
50	58.8	61.2	63.4	148.4	46.2	42.8	40.2	144.2
100	58.8	61.2	63.4	148.0	46.0	42.8	40.3	144.3
150	58.8	61.1	63.4	148.3	46.1	42.8	40.2	144.2
200	58.8	61.1	63.4	148.2	46.0	42.8	40.2	143.9

## Data Availability

The original contributions presented in this study are included in the article. Further inquiries can be directed to the corresponding authors.

## Abbreviations

The following abbreviations are used in this manuscript:

PCMs	phase change material
PEG	polyethylene glycol
CMWCNT	carboxylated multi-walled carbon nanotubes
KNFs	Kevlar nanofibers
CFK	CMWCNT/Fe^3+^/KNFs three-dimensional metal–organic network
MOFs	metal–organic framework
SEM	scanning electron microscope
FTIR	Fourier transform infrared
XRD	X-ray diffraction
TGA	thermogravimetric analysis
TC	thermal conductivity
DSC	differential scanning calorimetry
EDS	energy dispersive spectrometer
TEA	triethylamine
